# Cumin Prevents 17β-Estradiol-Associated Breast Cancer in ACI Rats

**DOI:** 10.3390/ijms22126194

**Published:** 2021-06-08

**Authors:** Farrukh Aqil, Jeyaprakash Jeyabalan, Radha Munagala, Iqbal Ahmad, David J. Schultz, Ramesh C. Gupta

**Affiliations:** 1 James Graham Brown Cancer Center, University of Louisville, Louisville, KY 40202, USA; farrukh.aqil@louisville.edu (F.A.); jp3pbiotech@gmail.com (J.J.); radha.munagala@gmail.com (R.M.); 2 Department of Medicine, University of Louisville, Louisville, KY 40202, USA; 3 Department of Ag Microbiology, Aligarh Muslim University, Aligarh 202002, India; iqbalahmad8@yahoo.co.in; 4 Department of Biology, University of Louisville, Louisville, KY 40208, USA; david.schultz@louisville.edu; 5 Department of Pharmacology and Toxicology, 580 S. Preston St. Rm 304E, Baxter II Research Building, University of Louisville, Louisville, KY 40202, USA

**Keywords:** *Cuminum cyminum*, cumin, breast cancer, estradiol, ACI rats

## Abstract

Breast cancer (BC) is a leading cause of cancer deaths in women in less developed countries and the second leading cause of cancer death in women in the U.S. In this study, we report the inhibition of E2-mediated mammary tumorigenesis by *Cuminum cyminum* (cumin) administered via the diet as cumin powder, as well as dried ethanolic extract. Groups of female ACI rats were given either an AIN-93M diet or a diet supplemented with cumin powder (5% and 7.5%, *w*/*w*) or dried ethanolic cumin extract (1%, *w*/*w*), and then challenged with subcutaneous E2 silastic implants (1.2 cm; 9 mg). The first appearance of a palpable mammary tumor was significantly delayed by both the cumin powder and extract. At the end of the study, the tumor incidence was 96% in the control group, whereas only 55% and 45% animals had palpable tumors in the cumin powder and extract groups, respectively. Significant reductions in tumor volume (660 ± 122 vs. 138 ± 49 and 75 ± 46 mm^3^) and tumor multiplicity (4.21 ± 0.43 vs. 1.16 ± 0.26 and 0.9 ± 0.29 tumors/animal) were also observed by the cumin powder and cumin extract groups, respectively. The cumin powder diet intervention dose- and time-dependently offset E2-related pituitary growth, and reduced the levels of circulating prolactin and the levels of PCNA in the mammary tissues. Mechanistically, the cumin powder diet resulted in a significant reversal of E2-associated modulation in ERα, CYP1A1 and CYP1B1. Further, the cumin powder diet reversed the expression levels of miRNAs (miR-182, miR-375, miR-127 and miR-206) that were highly modulated by E2 treatment. We analyzed the composition of the extract by GC/MS and established cymene and cuminaldehyde as major components, and further detected no signs of gross or systemic toxicity. Thus, cumin bioactives can significantly delay and prevent E2-mediated mammary tumorigenesis in a safe and effective manner, and warrant continued efforts to develop these clinically translatable spice bioactives as chemopreventives and therapeutics against BC.

## 1. Introduction

Breast cancer (BC) is the second leading cause of cancer-related deaths among women in the United States, with an estimated 281,550 new BC diagnoses and 43,600 new deaths in 2021 [1,2]. Twenty to forty percent of all BC cases metastasize after the original diagnosis, which drastically reduces the 5-year survival rate (to 22%) and this population of patients may benefit greatly from prophylactic intervention [3].

About 80% of all BC cases are estrogen receptor α (ERα)-positive [4] and ~50% of BC cases can be attributed to specific risk factors, including hormone exposure, gene mutations (e.g., BRCA1 and BRCA2), breast density, and obesity. The treatment for BC is usually multifaceted, including surgery, chemotherapy, radiation, and hormone therapy. However, these treatments burden patients with side effects that reduce therapy compliance and quality of life. In most patients where BC arises not from single gene mutations (or discrete combinations) [5,6], selective estrogen-receptor modulators (SERMs), e.g., tamoxifen and raloxifene, are approved for both the treatment and prevention of BC and decrease BC risk by about 35% [7]. However, tamoxifen is rarely used in clinics for prevention due to serious side effects such as endometrial cancer [8,9]. Raloxifene is less likely to cause endometrial cancer, but its use results in increased risk of cataracts and blood clots [7]. Notably, with the rise in obesity in the US (and world-wide), and the associated increase in BC risk [10], it is imperative to develop simple and effective strategies to prevent BC.

An accumulating body of scientific evidence indicates that many cancers are preventable, especially in relation to diet and nutrition, which are key factors in the modulation of cancer risk [11]. In fact, dietary habits are estimated to contribute to at least 35%, but perhaps as high as 70% for all human cancers [12,13]. By some accounts, more than 50% of BC patients and women at high risk of developing BC use some kind of alternative medicine [14,15] including “natural SERMs”, consisting largely of plant-based phytoestrogens [16] and other natural agents such as indole-3-carbinol and tea catechins [17]. However, the effectiveness of tea catechins and other plant bioactives are limited due to issues with oral bioavailability. Therefore, there is an ‘unmet need’ for identifying new plant bioactives that can be efficacious and safe for prolonged periods to prevent BC in high-risk women.

Natural products have been exploited from ancient times in the treatment of cancer. Cumin (*Cuminum cyminum* L.) is a member of the Apiaceae family, and its seeds are used mainly as a spice, constituting a major ingredient of most curry powders and many savory spice mixtures used in Indian cuisine and for flavoring curries, soups, sausages, and cakes, etc. In addition to its role as a flavoring agent, cumin has been shown to stimulate the activities of aryl hydroxylase, cytochrome p450s and N-demethylase in rats [18]. It also enhances the GST and suppresses the formation of aflatoxin-B1-induced DNA adducts formation [19]. The major phytochemical constituents of cumin are cuminaldehyde and cymene [20], and cumin seeds and the derived oil are regarded generally as safe (GRAS 2340 and GRAS 2343) [20]. Cumin seeds have also been utilized in polyherbal formulations due to estrogenic activity, an activity supported by research in ovariectomized rats [21]. Despite known and established pharmacological properties, very little work has been done on the chemopreventive effect of cumin.

The present study was undertaken to evaluate the chemopreventive potential of cumin seed powder as well as seed powder extracts in the 17ß-estradiol (E2)-induced mammary tumorigenesis August Copenhagen Irish (ACI) rat model. The female ovary-intact ACI rat provides a physiologically relevant and genetically defined animal model for studying diet–hormone interactions in mammary cancer development. As demonstrated by Shull et al. [22], this model is highly relevant for human BC as these estrogen-induced tumors exhibit aneuploidy, as do invasive ductal mammary carcinomas in women, and it is highly relevant to understanding the etiology and progression of breast cancer in humans, most particularly the luminal breast cancer subtypes. Further, the high-circulating E2 levels established in this model correlate with high E2 levels that increase the risk of BC in both premenopausal and postmenopausal women [23].

We previously demonstrated that aqueous and non-aqueous extracts of specific Apiaceae spices, including cumin, are protective against oxidative DNA adducts resulting from the redox cycling of 4-hydroxy E2 [24]. In the current study, we show that cumin seeds, administered as dietary powder and aqueous ethanolic extract of cumin seed powder, are highly effective against E2-mediated mammary tumors and explicate the mechanisms of action by the analysis of various cellular and molecular markers. These data further support our ultimate goal to develop cumin and other Apiaceae spices as chemopreventives against BC.

## 2. Results

### 2.1. Effect on Body Weight and Food Consumption

**Figure 1:**
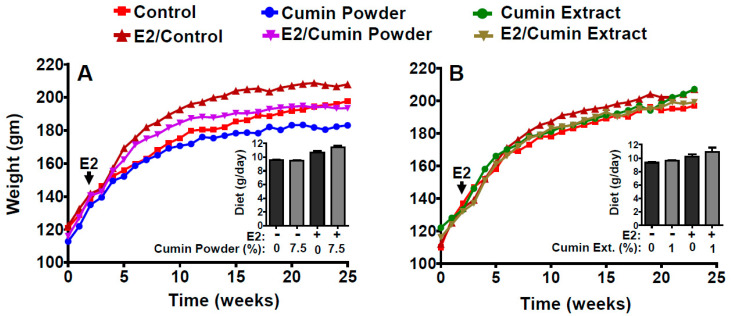
Effect of diet supplemented with cumin powder (7.5%, *w*/*w*) (**A**) and dried ethanolic cumin extract (1%, *w*/*w*) (**B**) on the body weight gain of the August Copenhagen Irish (ACI) rats. Inset in the left and right panels show the average daily diet consumption. Arrow in each figure shows the start of the 17ß-estradiol (E2) treatment.

The control animals fed an AIN93M diet (Supplementary Table S1) consumed an average of 9.55 ± 0.43 g/day/animal throughout the experiment. The consumption was increased to 10.71 ± 0.84 g/day/animal in the animals challenged with E2. The diets supplemented with cumin powder (5% and 7.5%, *w*/*w*) did not affect the diet intake when compared with the control, irrespective of E2 treatment. Also, there was no difference in the diet intake when comparing the two levels of cumin powder supplementation or when the diet was supplemented with 1% cumin extract, suggesting that all of the treatment diets were well tolerated (Figure 1).

There was no difference in the average body weight gain after three weeks of E2 implantation compared to the control (155.6 ± 12.7 versus 154.0 ± 4.5) (Figure 1A,B). However, a non-significant increase in the body weight gain (202.4 ± 8.6) of E2-treated animals was observed compared to the untreated control (191.5 ± 11.6) at the end of the study. The diet supplemented with 5% (data not shown) and 7.5% cumin powder offset the E2-associated gain in body weight at the end of the study (190.2 ± 10.7). The weight of the animals fed with 7.5% cumin powder was, in fact, somewhat lower than the rats fed the control diet (187 ± 9 g vs. 198 ± 15 g) (Figure 1).

### 2.2. Organ Weight

E2 treatment resulted in a slight to modest increase in the mammary tissue weight compared with vehicle treatment—1.2-fold after 3 weeks and 1.6-fold after 26 weeks; however, these increases were not significant (Table 1). The effect of E2 treatment was dramatic on the pituitary weight—a 2.4-fold increase after 3 weeks, reaching to 4.5-fold. These increases in the mammary and pituitary weights were significantly offset with the cumin powder-supplemented diet (Table 1). The weights for the liver and lung were not affected either with the E2 treatment or either of the diets.

### 2.3. Effect on Tumor Incidence, Latency, Multiplicity and Volume

**Figure 2:**
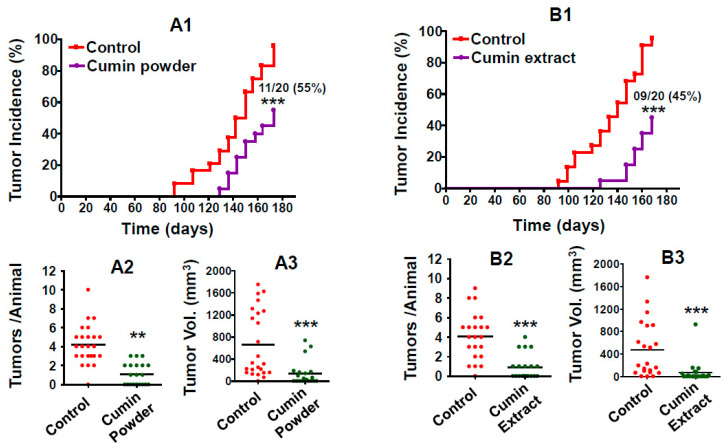
Effects of diet supplemented with cumin powder (7.5%, *w*/*w*) (**A**) and dried ethanolic cumin extract (1%, *w*/*w*) (**B**) on the following various mammary tumorigenesis indices: tumor incidence and latency (**A1**,**B1**), tumor multiplicity (**A2**,**B2**), and tumor volume (**A3**,**B3**). After acclimatization, ACI rats were subcutaneously grafted with 9 mg E2-containing silastic implants. Animals were palpated starting from week 12 and tumor incidence was recorded weekly and analyzed using a non-parametric log-rank test. Numbers denote the animals without tumor at the end of the study. Tumor multiplicity and tumor volumes were calculated at the time of euthanasia and analyzed using unpaired two-tailed Student’s *t*-test. Asterisk indicates significant difference between control diet versus cumin diet-fed animals. ** *p* < 0.01; *** *p* < 0.001.

Two studies were conducted to determine the effects of diets supplemented with either cumin powder (7.5%, *w*/*w*) (Study 1) or cumin extract (1%, *w*/*w*) (Study 2) on E2-mediated mammary tumorigenesis. After acclimation, the animals were randomized and provided either a control diet or experimental diet, and then 2 weeks later, the animals were treated with a subcutaneous E2 implant (n = 20 animals per group). The first palpable tumor in E2-treated animals on the control diet was detected after 93 days, and almost 96% of the animals exhibited one or more palpable mammary tumors at the end of the study. However, in the animals fed the diet supplemented with cumin powder, the first palpable mammary tumor was observed after 129 days of E2 treatment, and only 55% (11/20) of the animals had tumors at the end of the study (Figure 2A1). The differences in both the median latency and final mammary tumor incidence between E2-treated animals fed the control diet versus the experimental diet were highly significant (*p* < 0.001). Eleven of 24 rats in the E2-treated control group exhibited one or more tumors >1.5 cm in diameter in E2-induced mammary tumors, and therefore this study was terminated. In the second study, while the control animals showed 96% tumor incidence, the appearance of the first tumor was delayed by 35 days by an intervention with dried ethanolic cumin extract, and the tumor incidence was reduced to 45% (9/20) when the study was terminated (Figure 2B1).

Both cumin seed powder- and dried ethanolic cumin extract powder-supplemented diets significantly reduced the tumor burden and tumor multiplicity compared to the control diet. The average volume of the mammary tumors was almost five-fold lower with the cumin powder diet compared to the control diet (136 ± 50 versus 661 ± 123 mm^3^) (Figure 2A2). Only 22 mammary tumors were developed in the cumin diet group compared to 101 tumors in the control diet group, or on an average 1.1 ± 1.6 tumors/rat vs. 4.2 ± 0.4 tumors/rat (Figure 2A3). The differences observed in tumor multiplicity and tumor volume in the control vs. cumin-treated animals were both statistically significant (*p* < 0.01).

In the second study, with the dried ethanolic cumin extract powder intervention, a total of 90 tumors developed in the control group, while only 18 tumors were detected in the dried ethanolic cumin extract powder group or 0.8 tumor/rat versus 4 tumors/rat in the control (Figure 2B3); the tumor volume was also significantly reduced (474 vs. 75 mm^3^) (Figure 2B2). The reductions in tumor volume (79% and 85%) and multiplicity (74% and 78%) were very similar between the cumin powder and dried ethanolic cumin extract powder, respectively, compared to the control.

### 2.4. Effect on Mammary Cell Proliferation

**Figure 3:**
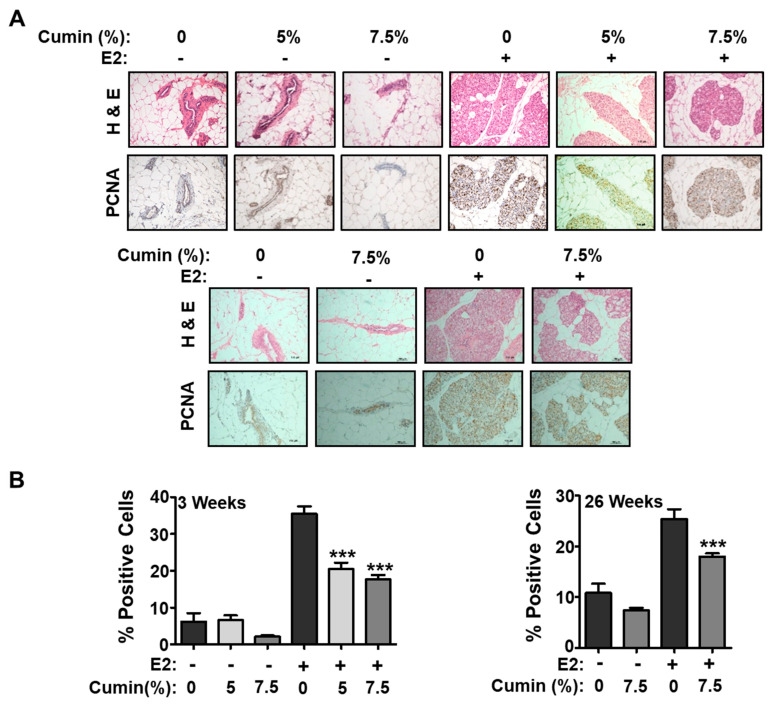
Effect of cumin diet on 17ß-estradiol (E2)-mediated mammary cell proliferation in the absence and presence of E2 treatment. (**A**) Representative photomicrographs (20× magnification) of normal and hyperplastic mammary tissue following immuno-histochemical staining for proliferating cell nuclear antigen (PCNA) after 3 weeks (left panel) and after 26 weeks (right panel). (**B**) Bar diagrams show the percentage (average ± SD) of deeply stained cells for PCNA in mammary tissues (n = 5). *** *p* < 0.001.

PCNA has proven to be a useful marker to evaluate cell proliferation. Mammary cell proliferation was analyzed by immunohistochemistry for the proliferating cell nuclear antigen (PCNA). Figure 3A shows representative images of PCNA-stained tissues and the effect of cumin powder intervention on the cell proliferation. No difference was observed in the animals fed with the control diet and the diet supplemented with cumin powder (5% or 7.5%, *w*/*w*, respectively). The mammary cell proliferation in the animals challenged with E2 showed an increase of about five-fold as analyzed by the counting of darkly stained cells. The cumin intervention inhibited the E2-mediated increase in the cell proliferation in a dose-dependent manner, and decreased the PCNA levels by almost 50% (Figure 3).

### 2.5. Effect on Circulating E2 and Prolactin Levels

**Figure 4:**
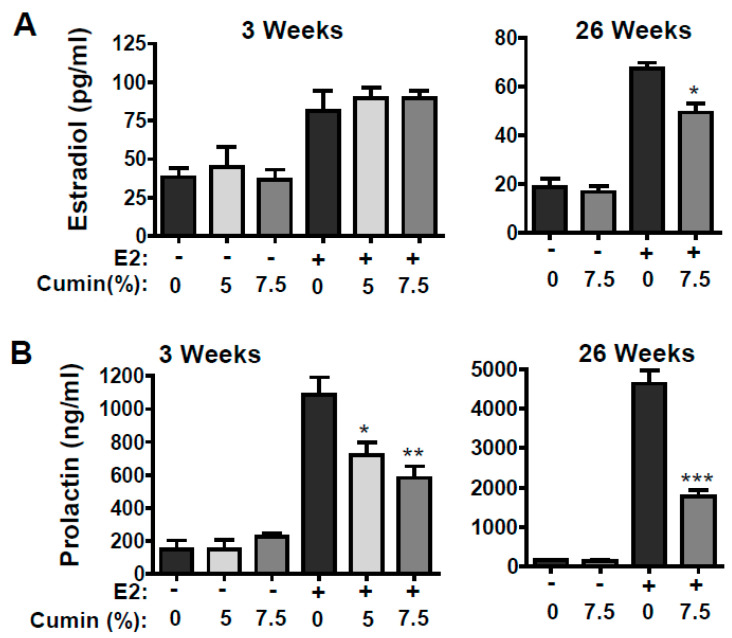
Effect of cumin powder diet on circulating 17ß-estradiol (E2) and prolactin. Time- (3 and 26 weeks, 7.5%, *w*/*w*) and dose-dependent (5% vs. 7.5%, *w*/*w* at 3 weeks) effect of cumin diet on circulating E2 levels (**A**) or prolactin levels (**B**) was measured in the blood plasma of the animals and compared with control animals. Data represent mean ± SD (n = 6). * *p* < 0.05, ** *p* < 0.01, *** *p* < 0.001.

It is well documented that higher levels of E2 stimulate mammary cell proliferation are associated with breast cancer risk. In this study, we examined the associations of mammary tumorigenesis with circulating estradiol. The basal blood plasma E2 levels in the animals fed with the control diet were found to be 38.3 ± 15.5 pg/mL. The basal E2 levels were not affected with the diet supplemented with cumin powder at 5% as well as 7.5% for 3 weeks. However, the E2 levels were increased significantly (86 pg/mL; *p* < 0.01) after 3 weeks of E2 treatment alone. Dietary cumin did not affect this increase in the E2 levels during this period; however, at the end of the study (26 weeks), the plasma E2 levels were significantly reduced (Figure 4A).

Unlike E2 levels, the effects of E2 treatment and dietary cumin on plasma prolactin levels were more dramatic. The basal levels of circulating prolactin were found to range from 150 ng/mL at 3 weeks to 1090 ± 259 ng/mL of E2 treatment. The cumin diet decreased the levels in a dose-dependent manner to 720 ± 155 and 583 ± 157 by 5% and 7.5% cumin-supplemented diets, respectively (Figure 4B), at 3 weeks. The animals in the tumor time point receiving E2 treatment showed a dramatic increase in the plasma prolactin level (4629 ± 839), which was very significantly mitigated with dietary cumin (1767 ± 405 ng/mL; *p* < 0.001) (Figure 4B) at 26 weeks.

### 2.6. Effect on Expression of Select miRNAs

**Figure 5:**
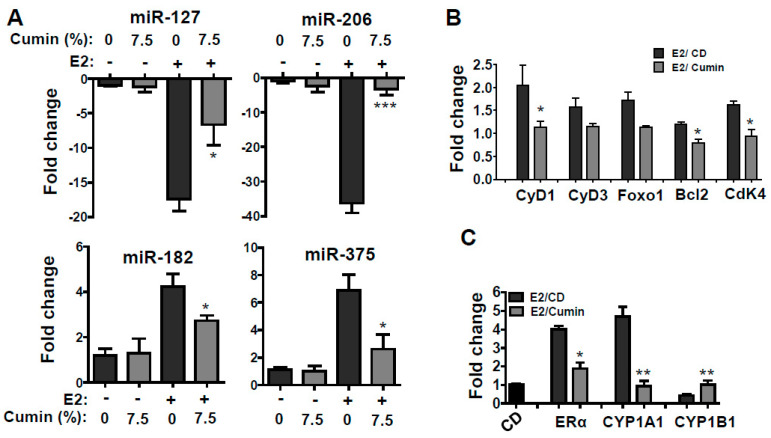
Effect of dietary cumin seed powder (7.5%, *w*/*w*) on select microRNAs (**A**), their downstream targets (**B**) and 17ß-estradiol (E2)-related markers (**C**) in E2-mediated mammary tumorigenesis. Total RNA was used to assess relative gene expression using the differences in normalized Ct (^ΔΔ^Ct) method after normalization to 18s rRNA. Fold changes were calculated by ^2-ΔΔ^Ct. Experiments were performed in triplicate for each data point. Data represent mean ± SD of fold change in mRNA expression relative to the untreated control fed with AIN93M control diet (CD) of five animals. Statistical significance was analyzed by Student’s *t* test where * *p* < 0.05; ** *p* < 0.01; *** *p* < 0.001.

We established the role of micro RNAs (miRNAs) in the development of E2-mediated mammary tumorigenesis [25] and showed that at least 33 miRNAs were significantly modulated in the ACI rat model of mammary tumorigenesis [26]. In this study, we analyzed the effect of cumin-supplemented diet on select miRNAs, viz., miR-127, -206, -182, and -375. As depicted in Figure 5A, in the absence of E2, there was no difference in the expression of these miRNAs between the control diet and the diet supplemented with 7.5% cumin powder. However, the expression levels of miR-182 and miR-375 were found to be significantly upregulated with E2 treatment, while the expression levels of miR-127 and miR-206 were significantly downregulated, corroborating our previous findings [26]. Importantly, these changes in the miRNA expression levels were favorably modulated by the treatment with 7.5% cumin seed powder (Figure 5A).

To confirm the cellular effects of miRNAs modulation, the expression levels of the downstream targets (cyclin D1, cyclin D3, Foxo1, Bcl2, and Cdk4) were also assessed. E2 treatment increased the expression of each downstream target and dietary intervention with cumin powder, and significantly reversed the E2-associated expression of these mRNA targets (Figure 5B). This supports the notion that the modulation of select miRNAs (miR-127, -206, -182, and -375) is associated with E2-mediated mammary tumorigenesis, and that dietary cumin, at least in part, inhibits mammary tumorigenesis by favorably modulating the expression of these miRNAs.

### 2.7. Effect on Expression Levels of ERα, CYP1A1 and CYP1B1

Since cytochrome P450s play major roles in the metabolism of E2, we analyzed the related molecular markers by RT-PCR and expressed the results as fold change. A diet supplemented with 7.5% cumin (*w*/*w*) did not affect the mRNA levels of estrogen receptor alpha (ERα) and select cytochrome P450s (CYP1A1 and CYP 1B1) relative to the controls. However, the mRNA levels were significantly modulated by the E2 treatment. While E2 enhanced the levels of ERα and CYP1A1 by about four- and six-fold, respectively, the levels of CYP1B1 were reduced by over three-fold. Interestingly, the effect of E2 treatment in modulating the mRNA of these molecules was almost completely diminished by the cumin intervention (*p* < 0.05) (Figure 5C).

### 2.8. Systemic Toxicity

**Figure 6:**
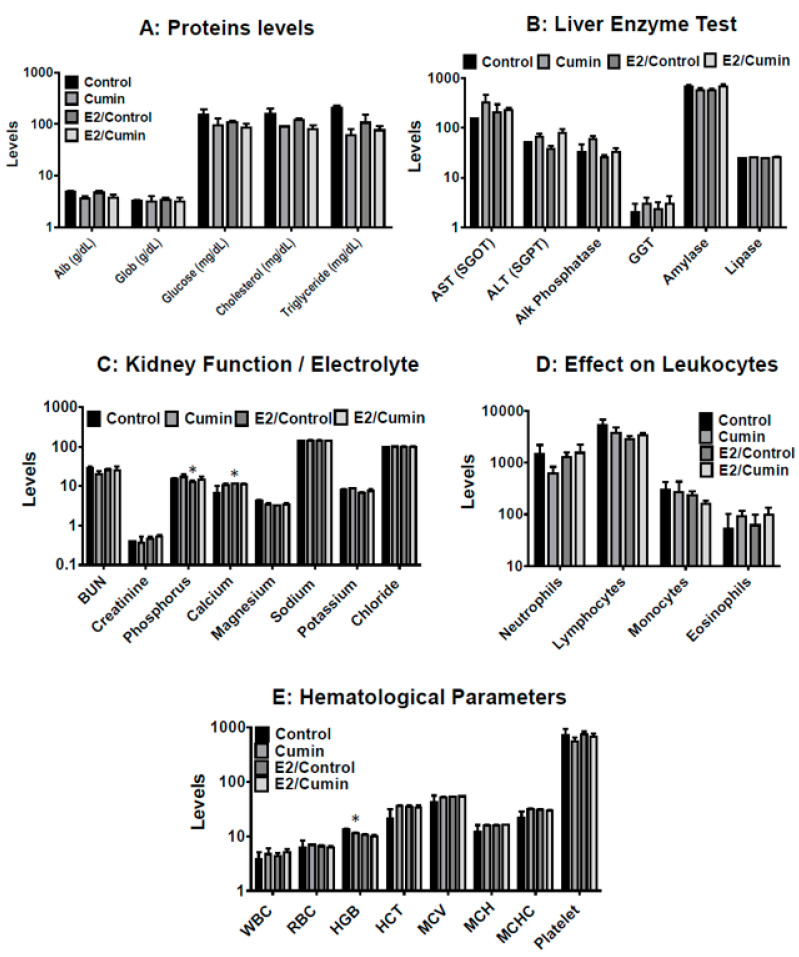
Effect of dietary cumin on the liver and kidney function enzymes, hematopoietic parameters and biochemical parameters in ACI rats. ACI rats (5–6 weeks old) were provided control diet (AIN 93M) or diet supplemented with cumin seed powder (7.5%, *w*/*w*) in the absence and presence of E2 for 26 weeks. Animals were euthanized and blood plasma was analyzed for (**A**) protein levels, (**B**) liver enzyme test, (**C**) kidney functions/electrolyte, (**D**) effect on leukocytes and (**E**) hematological parameters. Data represent mean ± SD. Student’s t-test was used for statistical analysis. * *p* < 0.05.

The effect of the E2 treatment and cumin diet was analyzed on liver and kidney function enzymes, hematological parameters and biochemical parameters. No difference in the diet consumption, body weight gain or any sign of gross toxicity was observed during the seven-month treatment time, indicating a lack of gross toxicity. Furthermore, neither dietary cumin nor E2 treatment significantly affected the liver and kidney function enzymes compared to the control diet; no significant effects of these treatments were observed on the hematological or biochemical parameters (Figure 6). Together, these data indicate that dietary administration of cumin powder over a period of 26 weeks was well tolerated.

### 2.9. Phytochemicals Detected in Cumin Extract by GC/MS Analysis

**Figure 7:**
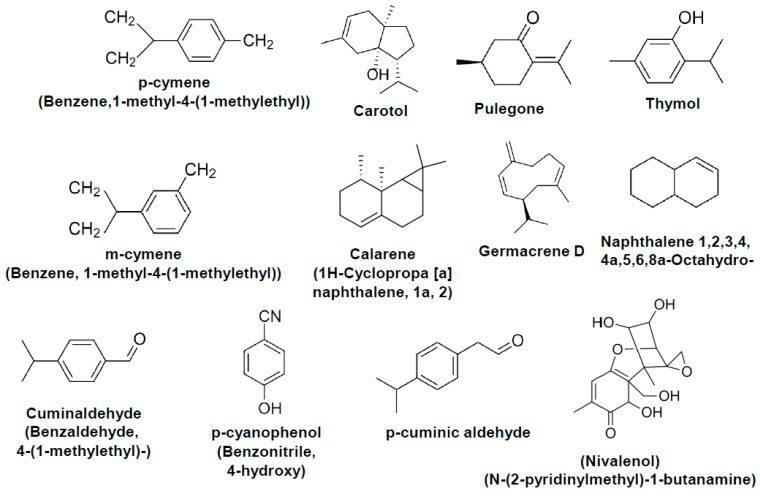
Chemical structure of phytocompounds identified in cumin. Ethanolic cumin extract was dissolved in methanol and analyzed by GC-MS to identify the chemical constituents on a GCD 1800A Hewlett Packard apparatus coupled with a HP-1 column. Helium was used as a carrier gas at the rate of 1 mL/min. Identification of major compounds was carried out on the basis of exact mass and compared with MS reference database of NBS75K.

To identify potential bioactives in cumin seeds, we analyzed the dried ethanolic cumin seed extract by GC-MS analysis. This analysis largely detected the presence of cuminaldehyde, p-cuminic aldehyde and terpenes (m-cymene, p-cymene, caratol, calarene, germacrene D, and nivalenol). The cumin extract also revealed the presence of pulegone, naphthalene, p-cyanophenol, as well as thymol, a monoterpene phenol derivative of cymene (Figure 7). The relative peak area is shown in the Supplementary Tables S1–S4.

## 3. Discussion

There has been a growing body of interest in the utilization of plant bioactives against various cancers in the recent years. With over one million new cases each year globally, BC is the most common malignancy in women, and the most common risk factor for BC is through hormone-related pathways and higher circulating E2 levels that increase the BC risk in postmenopausal women [23].

In the current study, we demonstrate the efficacy of *Cuminum cyminum* (cumin) to delay tumor indices, including tumor latency, incidence, multiplicity and tumor burden in the E2- mediated ACI rat mammary tumorigenesis. To more completely address the potential efficacy of cumin to modulate BC outcomes, we addressed a number of research goals, including (i) determining the chemopreventive efficacy of the Apiacea spice, cumin; (ii) comparing the anticancer efficacy of cumin seed powder versus dried ethanolic cumin seed extract powder; (iii) determining the effect of cumin on select microRNAs and molecular pathways associated with E2-induced mammary tumorigenesis; (iv) assessing toxicity due to E2 treatment or cumin intervention; and (v) determining the phytochemical signature of the cumin used in these studies.

The significant effect of cumin in the reduction in tumor indices was achieved without affecting the body weight gain or causing systemic toxicity. As in our previous chemoprevention studies with berries [27,28], we used cumin intervention via the dietary route due to ease of administration. Since the spice doses tested in our previous studies and this study are not clinically translatable, we also tested dried aqueous ethanolic extract of cumin seed powder administered dietarily at a comparable level to whole cumin seed powder (7.5%).

Many small molecules that show effectiveness at inhibiting in vitro cancer cell growth, either produce adverse reactions in pre-clinical studies or do not show effectiveness in clinical studies. In most of the cases, toxicity is the primary limitation. Health-related issues in the animals, such as loss of body weight, change in hair coats, limping, and reduction in diet intake, are established predictors of disease progression. While the animals did not show any difference in the consumption of the cumin powder diet (7.5% *w*/*w*) and cumin extract diet (1% *w*/*w*), the body weight gain of age-matched ACI rats was somewhat lower, though not significantly different in the cumin seed powder diet-fed animals compared to the control diet. In addition, we [27,28,29] and others [22] have shown that E2 induces mammary cell proliferation in female rats. The current study also corroborated the previous studies and shows about a 20% increase after 3 weeks and about a 40% increase at the time of euthanasia in the mammary tissue weights. The increase in mammary weight was irrespective of dietary intervention.

While there was no difference in the liver and mammary weight of the animals fed with the cumin-supplemented diet, the weight of the pituitary gland was significantly reduced by the dietary cumin powder in a dose-dependent manner (Table 1). Interestingly, the effect of dietary cumin powder on pituitary weight is well correlated with the circulating prolactin levels. Since increased circulating levels of prolactin have been associated with mammary tumorigenesis [30,31,32], it suggests a correlation between the significant cumin-mediated reduction observed in the prolactin level and tumor inhibition. We have also noted in our previous studies that berry anthocyanidins have SERM-like activities, and therefore play a role in the reduction in E2 levels [28]. Contrary to our previous studies with blueberry, black raspberry and jamun [27,28], the dietary cumin did not significantly affect the circulating E2 levels or the reduce mammary weight, further suggesting that cumin-mediated inhibition of mammary tumors, at least in part, is attributed to the mitigation of increased circulating prolactin levels. This mechanism would be consistent with previous work that indicates that tamoxifen inhibits estrogen-induced mammary tumors via its interaction with the pituitary gland without altering the circulating E2 levels [33].

Treatment with cumin, whether whole seed powder or dried ethanolic extracts of seed powder, should have strong efficacy. In comparing the effects of the two treatments, the tumor latency or the delayed appearance of the first tumor was similar between the intervention with cumin powder and cumin extract diet. However, tumor incidence was somewhat better with the cumin extract (45%, 9 of 20 rats with tumors) compared with the cumin powder (55%, 11 of 20 rats with tumors). The inhibition of tumor multiplicity and reduction in tumor volume were very similar in both of the interventions. Based on the allometric calculations, a human equivalent dose of about 81 mg/kg of cumin dried extract is needed compared to a 630 mg/kg dose of cumin seeds (Supplementary Table S5). Thus, a clinically relevant dose may be easier to achieve dietarily using ethanolic extracts compared to whole seed powders.

Estrogen receptor-mediated gene expression leads to mammary cell proliferation and has been postulated to play a significant role in E2-induced mammary tumorigenesis. In this model of mammary tumorigenesis, we have shown that Apiaceae spices, including Anise, caraway and fennel, demonstrate strong antiproliferative activity [29]. Similar to the previous study, E2 significantly induced proliferation in mammary tissues, which was dose-dependently diminished by the cumin powder intervention after 3 weeks as well as after 26 weeks. E2 primarily affects Erα, which is required for the growth of mammary glands. We noted that the cumin powder intervention reduces the ERα and cyclin D1, suggesting the anti-estrogenic nature of cumin phytochemicals. The abnormal expression of cyclin D1 has been well-documented in human BC and overexpressed in more than 50% of breast cancers [34].

E2 induces oxidative damage by its metabolites 4-hydroxy-estradiol [35]. In this study, we tested the effect of cumin intervention on the levels of cytochrome p450s, and we show that cumin intervention significantly reversed the E2-mediated changes in CYP1A1 and CYP1B1, which corroborated our previous data in which we demonstrated the free radical scavenging activity of several Apiaceae spices [24].

The miRNAs are a class of small non-coding RNAs. These are post-transcriptional gene regulators and have a size of 18–23 nt in length. In the last decade, several studies have established a correlation between the modulation of select miRNAs and the regulation of BC initiation and progression. In a study of 76 BC and 10 normal breast samples, 29 miRNAs were found significantly dysregulated [36]. In the ACI rat model, we have also identified 33 miRNAs that were highly modulated in the presence of estradiol [26]. We chose four highly modulated miRNAs (miR-127, -206, -182, and -375) to determine the effect of cumin bioactives. A cumin diet reversed the E2-associated up- or downregulation of these molecules and reversed the downstream target effects of these miRNA, suggesting that the effects on miRNAs are further broad and might have impacted the inhibition of mammary tumor development.

No signs of gross toxicity were observed with the diets supplemented with the cumin seed powder nor dried ethanolic extracts cumin seed powder, based on body weight gains, diet consumption, hair coat quality and movement of the animals. Further, no systemic toxicity was observed with the cumin diet at the highest dose (7.5%) tested, based on insignificant effects on the liver and kidney function enzymes, and hematopoietic and biochemical parameters over the long duration (26 weeks) of the study, consistent with results we have noted with other Apiaceae spice (Caraway) and jamun berry [27,29]. A lack of systemic toxicity based on blood chemistry further suggest the tolerance of cumin at the tested doses.

Based on the GC-MS analysis of the ethanolic extracts of cumin seed powder, various terpenoids and other phytochemicals with known anticancer activity were detected. We have previously reported the BC chemopreventive activity of various phenolics and flavonoids including berry anthocyanidins [28,37], and ellagic acid [38,39]. In this study, both cumin powder and aqueous ethanolic cumin extract demonstrate anti-cancer effects against E2-mediated mammary tumorigenesis, similar to berry phenolics. The major constituents of cumin are cuminaldehyde, cymene and other terpenoids [20]. In this study, we found an abundance of terpenes (m-cymene, β-cymene, caratol, carvacrol, germacrene D, and nivalenol) in the ethanolic extract of cumin seed powder. We also detected thymol, a monoterpene phenol derivative of cymene. Cymene is very effective in promoting in vitro cytotoxic effects on HER2^+^ and RH^+^/HER2^–^ BC, and showed significant cytotoxicity against BT474 BC cells [40]. Similarly, carvacrol, germacrene D and other chemicals have been shown to have strong antiproliferative activity against BC cells [41,42].

In summary, cumin is a rich source of phytochemicals, with breast cancer chemo-prevention potential. Our data suggest the inhibition of mammary tumors by cumin bioactives could be due to the reversal of E2-mediated modulations in cell proliferation and E2-related markers, including the reduction in circulating prolactin levels. Finally, the inhibition, by only 1% cumin extract via the diet and lack of chronic toxicity demonstrated in this study, further suggests the feasibility of cumin use and its potential to develop as a cancer chemopreventive agent.

## 4. Materials and Methods

### 4.1. Chemicals

The silastic tube of 2.0 mm internal diameter and 3.2 mm outer diameter was purchased from Allied Biomedical, Inc. (Ventura, CA, USA). Silicone adhesive was purchased from Factor II, Inc. (Lakeside, AZ, USA), and 17ß-estradiol was purchased from Steraloids, Inc. (Newport, RI, USA). The antibodies (ERα, progesterone receptor, 1A1 and 1B1) were purchased from Santa Cruz Biotechnology (Santacruz, CA, USA), cyclin D1, Cdk4 from Abcam (Cambridge, MA, USA) and β-actin was purchased from Sigma-Aldrich (St. Louis, MO, USA). All other chemicals used were of analytical grade.

### 4.2. Diet Preparations

Cumin (*Cuminum cyminum*) seeds (Frontier Herbs, Danbury, CT, USA) were powdered and extracted three times with 80% ethanol, filtered through 0.22 µM Whatman filter paper, and dried under vacuum. Control purified AIN-93M diet was purchased as pellets from Harlan Teklad Inc. (Madison, WI, USA). Pellet diet supplemented with cumin powder (5% and 7.5% *w*/*w*) and equivalent dried ethanolic cumin extract powder (1%, *w*/*w*) were custom made matching caloric content to the AIN diet from Harlan Teklad. Diets were stored vacuum sealed at 4 °C until use.

### 4.3. Animal Study

**Figure 8:**
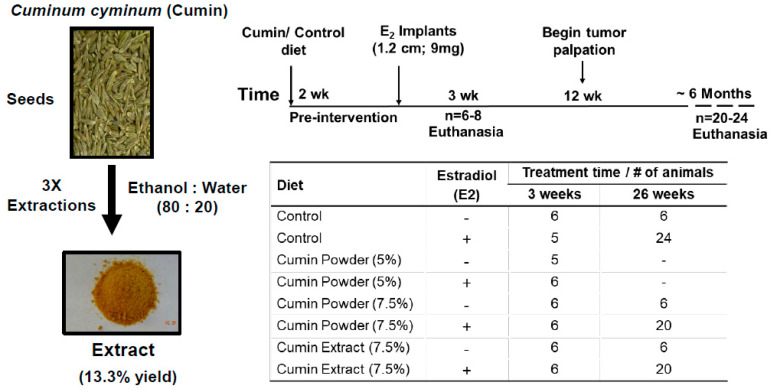
Ground *Cuminum cyminum* (cumin) seeds powder and derived dried ethanolic extract was used in this study. Schematic representation of animal study plan; table shows the animal study design used in the study.

Five to six-week-old female ACI rats were purchased from Harlan Sprague–Dawley (Indianapolis, IN). All animals were maintained according to the Institutional Animal Care and Use Committee (IACUC) guidelines (IACUC # 13008; approval date: 1 August 2013) of the University of Louisville, Louisville, KY, USA. The rats were housed in cages and received food and water ad libitum. Three studies were conducted. In study 1, we determined the dose response of cumin powder diet. After one week of acclimatization, animals were randomized into 6 groups and received either control diet or diet supplemented with cumin powder (5% and 7.5, *w*/*w*) (Figure 8). Two weeks later, animals in E2 treatment groups were subcutaneously grafted with 1.2 cm silastic implant containing 9 ± 0.2 mg E2, as described previously [32]. After three weeks, all animals were euthanized and blood and various organs were collected. The mammary tissues were analyzed for proliferation marker (PCNA), and blood was analyzed for circulatory prolactin and estradiol levels. Since cumin powder diet showed dose-dependent response with higher activity at 7.5% diet, tumor study was conducted using 7.5% cumin diet.

In the second study, animals were randomized into 4 groups and treated with either control diet or diet supplemented with cumin powder (7.5% *w*/*w*). Body weights and diet consumption were assessed weekly for all groups until termination. Starting from week 12 the animals challenged with E2 were palpated for mammary tumors weekly to determine tumor incidence and development. When the tumor incidence in E2/control diet group reached ~90%, animals in all groups were euthanized by CO_2_ asphyxiation, blood was drawn and the number of tumors per rat were determined. Mammary (both tumor and normal tissues), liver, lung, pituitary gland and other select organs were collected and weighed. A small piece of each tissue was transferred to 10% buffered formalin for histopathological analysis and immunohistochemistry. The remaining tissues were snap-frozen in liquid nitrogen and stored at −80 °C for future analyses.

Study 3 was patterned after the second study and the animals provided diet supplemented with 1% dried ethanolic cumin extract, which equates to 7.5% cumin powder diet based on the extraction efficiency of about 13.3%. The total calories, fiber content and other components were matched with the control AIN 93M diet.

### 4.4. Assessment of Cell Proliferation

A portion of the excised mammary tissue was stored in 10% buffered formalin and transferred to 70% ethanol the next day for histopathologic analyses. The formalin-fixed tissue was embedded in paraffin, and 4–5 μm sections were cut. Paraffin-embedded sections of the mammary tissue were stained with H&E (hematoxylin and eosin) and PCNA levels were quantified using Zymed PCNA kit (Invitrogen Co., Carlsbad, CA, USA) as described previously [27,43]. Deeply stained cells were scored blinded by two pathologists. Under each field, 100 cells were counted and 10 such fields were scored and average values are presented. Images were acquired using Nikon NIS-Elements software.

### 4.5. Circulating E2 and Prolactin Analysis

Circulating serum E2 was analyzed in blood samples by electrochemiluminescent detection using the Roche E170 immunoassay analyzer at the university hospital’s clinical chemistry facility as described [28]. All samples were assayed in triplicate. Levels of circulating prolactin, secreted by the pituitary prolactinomas, were determined in plasma samples using the Rat Prolactin EIA kit (Alpco Diagnostics, Windham, NH, USA), as per manufacturer’s instructions.

### 4.6. RNA Isolation, qRT-PCR and Analysis of Gene Expression

RNA from mammary tissue was isolated using the Trizol method (Invitrogen). Briefly, mammary tissue was suspended in Trizol at 4 °C and homogenized with a handheld polytron homogenizer at maximum speed. The tissue homogenate was sequentially extracted with chloroform, and the aqueous phase was precipitated with ice-cold isopropanol. The quality of RNA was ascertained by gel electrophoresis and quantitated using NanoDrop (NanoDrop Technologies). RNA (3 ng) was used for qRT-PCR using one-step qPCR kit (QuantaBio, Beverly, MA, USA).

Primers for qRT-PCR were designed across the exon boundary using Primer Express 3.0 software (Applied Biosystems) and synthesized by Integrated DNA Technologies, Inc. as described [26]. The sequences of the forward and reverse primers are the same as described previously [27]. Quantitative PCR utilized a 7500 fast real-time PCR system (Applied Biosystems). Amplification conditions utilized 50 °C for 2 min, DNA polymerase activation at 95 °C for 10 min, followed by 40 cycles at 95 °C for 15 s and 60 °C for 1 min. At least four biological samples were used for all qRT-PCR reactions and each sample was analyzed in triplicate for each gene tested.

Gene expression analysis utilized the relative quantification (^ΔΔ^*C*_T_) method as described [44]. The results are represented as relative fold change, which is the average fold change among the biological replicates (*n* = 4–6 per group) and represents the biological variation within a specific group.

### 4.7. Toxicity Assessment

Hematological parameters were analyzed using whole blood by Cell Dyn 3500 hematology analyzer (Abbott laboratories, Santa Clara, CA, USA). Blood serum was used to analyze the liver and kidney enzyme function. An ion-selective electrode was used for electrolyte analysis and built-in spectrophotometric techniques were used for analyses of other biochemical parameters by an automated AU640 chemistry analyzer (Beckman Coulter, Inc., Brea, CA, USA).

### 4.8. Analysis of Cumin Extract by GC–MS

The cumin extract was dissolved in methanol and subjected to GC-MS analysis in order to identify the chemical constituents on a GCD 1800A Hewlett Packard apparatus coupled with a HP-1 column (30 m × 0.25 mm × 0.25 μm). The temperature program was set at an initial temperature of 100–250 °C at a rate of 10 °C/min withhold time at 250 °C for 3 min, and final temperature of 250–280 °C at a rate of 30 °C/min with a hold time of 2 min at 280 °C. Helium was used as a carrier gas at the rate of 1 mL/min.

Identification of major compounds was carried out on the basis of exact mass and compared with MS reference database of NBS75K at the Sophisticated Analytical Instrument Facility of the Indian Institute of Technology Bombay, Mumbai.

### 4.9. Statistical Analyses

Statistical analysis of control and treatment group was carried out as follows: Averages of target parameters are indicated with standard deviation and *p*-values determined by Student’s *t*-test. Tumor multiplicity was analyzed using the negative binomial regression model with logarithmic link. Tumor latency was defined as the number of days of E2 treatment before the appearance of the first palpable mammary tumor, based on weekly examination. Tumor latency was determined using Kaplan–Meier survival estimates and plots. Comparisons of latency among groups were made using the log-rank test. When the overall log-rank test was significant, pairwise log-rank tests were done with *p*-values adjusted using the Bonferroni method. Differences in average number of mammary tumors were assessed using the Kruskal–Wallis ANOVA with Dunn’s post-hoc tests (GraphPad Prism, version 4.0 for Windows, GraphPad Software, San Diego, CA, USA). A *p*-value < 0.05 was considered significant in all cases. These data are presented as mean ± SD.

## Figures and Tables

**Table 1:** Effect on body, organs, and tissue weights by cumin intervention.

Time Point	Diet	E2	Organ/Tissue Weights (Mean ± SD)				
			Body wt (g)	Liver (g)	Lung (g)	Mammary (g)	Pituitary (mg)
3 weeks	Control	−	154.0 ± 4.5	4.9 ± 0.2	0.9 ± 0.2	4.3 ± 0.4	8.1 ± 1.3
	Control	+	155.6 ± 12.7	7.4 ± 0.7	1.0 ± 0.2	5.2 ± 0.6	19.2 ± 3.5
	5% Cumin	−	153.5 ± 9.4	5.5 ± 0.2	1.0 ± 0.1	3.7 ± 0.9	8.6 ± 0.4
	5% Cumin	+	159.0 ± 13.9	7.4 ± 0.4	1.0 ± 0.1	5.0 ± 0.5	15.8 ± 2.3 *
	7.5% Cumin	−	153.5 ± 9.4	5.9 ± 0.3	1.0 ± 0.1	3.6 ± 0.1	8.1 ± 1.3
	7.5% Cumin	+	151.3 ± 6.2	7.8 ± 0.4	0.9 ± 0.2	4.0 ± 1.0 *	9.3 ± 1.8 ***
26 weeks	Control	−	191.5 ± 11.6	5.9 ± 0.4	1.1 ± 0.2	4.4 ± 1.2	11.1 ± 1.0
	Control	+	202.4 ± 8.6	6.9 ± 0.6	1.0 ± 0.1	7.0 ± 0.8	49.9 ± 11.4
	7.5% Cumin	−	182.5 ± 14.6	5.7 ± 0.6	0.9 ± 0.1	5.2 ± 0.7	8.4 ± 1.5
	7.5% Cumin	+	190.2 ± 10.7	6.9 ± 0.6	1.0 ± 0.2	7.0 ± 0.8	38.4 ± 7.5 *

Asterisks show significant difference between E2/cumin to E2-treated control animals at * *p* < 0.05; *** *p* < 0.001. E2, 17β-estradiol.

## Data Availability

Not applicable.

## Supplementary Materials

The following are available online at https://www.mdpi.com/article/10.3390/ijms22126194/s1.

## References

[1] Siegel R.L., Miller K.D., Goding Sauer A., Fedewa S.A., Butterly L.F., Anderson J.C., Cercek A., Smith R.A., Jemal A. (2020). Colorectal cancer statistics, 2020. CA Cancer J. Clin..

[2] Siegel R.L., Miller K.D., Fuchs H.E., Jemal A. (2021). Cancer Statistics, 2021. CA Cancer J. Clin..

[3] Howlader N., Noone A., Krapcho M., Miller D., Bishop K., Altekruse S.F., Kosary C.L., Yu M., Ruhl J., Tatalovich Z. SEER Cancer Statistics Review, 1975–2013.

[4] Dai X., Xiang L., Li T., Bai Z. (2016). Cancer Hallmarks, Biomarkers and Breast Cancer Molecular Subtypes. J. Cancer.

[5] Fanfani V., Zatopkova M., Harris A.L., Pezzella F., Stracquadanio G. (2020). Dissecting the heritable risk of breast cancer: From statistical methods to susceptibility genes. Semin. Cancer Biol..

[6] Ellsworth D.L., Turner C.E., Ellsworth R.E. (2019). A Review of the Hereditary Component of Triple Negative Breast Cancer: High- and Moderate-Penetrance Breast Cancer Genes, Low-Penetrance Loci, and the Role of Nontraditional Genetic Elements. J. Oncol..

[7] Vogel V.G. (2011). Update on raloxifene: Role in reducing the risk of invasive breast cancer in postmenopausal women. Breast Cancer.

[8] Nichols H.B., DeRoo L.A., Scharf D.R., Sandler D.P. (2015). Risk-benefit profiles of women using tamoxifen for chemoprevention. J. Natl. Cancer Inst..

[9] Chen J.Y., Kuo S.J., Liaw Y.P., Avital I., Stojadinovic A., Man Y.G., Mannion C., Wang J., Chou M.C., Tsai H.D. (2014). Endometrial cancer incidence in breast cancer patients correlating with age and duration of tamoxifen use: A population based study. J. Cancer.

[10] Hillers-Ziemer L.E., Arendt L.M. (2020). Weighing the Risk: Effects of Obesity on the Mammary Gland and Breast Cancer Risk. J Mammary Gland. Biol. Neoplasia.

[11] Anand P., Kunnumakkara A.B., Sundaram C., Harikumar K.B., Tharakan S.T., Lai O.S., Sung B., Aggarwal B.B. (2008). Cancer is a preventable disease that requires major lifestyle changes. Pharm. Res..

[12] Donaldson M.S. (2004). Nutrition and cancer: A review of the evidence for an anti-cancer diet. Nutr. J..

[13] Willett W.C. (1995). Diet, nutrition, and avoidable cancer. Environ. Health Perspect..

[14] Dale L.C., Gotay C.C. (2012). The Relationship between Complementary and Alternative Medicine Use and Breast Cancer Early Detection: A Critical Review. Evid. Based Complement. Altern. Med..

[15] Wanchai A., Armer J.M., Stewart B.R. (2010). Complementary and alternative medicine use among women with breast cancer: A systematic review. Clin. J. Oncol. Nurs..

[16] Oseni T., Patel R., Pyle J., Jordan V.C. (2008). Selective estrogen receptor modulators and phytoestrogens. Planta Med..

[17] Wang J., Jiang Y.F. (2012). Natural compounds as anticancer agents: Experimental evidence. World J. Exp. Med..

[18] Sambaiah K., Srinivasan K. (1989). Influence of spices and spice principles on hepatic mixed function oxygenase system in rats. Indian J. Biochem. Biophys..

[19] Kleiner H.E., Vulimiri S.V., Miller L., Johnson W.H., Whitman C.P., DiGiovanni J. (2001). Oral administration of naturally occurring coumarins leads to altered phase I and II enzyme activities and reduced DNA adduct formation by polycyclic aromatic hydrocarbons in various tissues of SENCAR mice. Carcinogenesis.

[20] Sahana K., Nagarajan S., Rao L.J.M., Preedy V.R., Watson R.R., Patel V.B. (2011). Cumin (*Cuminum cyminum* L.) Seed Volatile Oil: Chemistry and Role in Health and Disease Prevention. Nuts and Seeds in Health and Disease Prevention.

[21] Shirke S.S., Jagtap A.G. (2009). Effects of methanolic extract of *Cuminum cyminum* on total serum cholesterol in ovariectomized rats. Indian J. Pharmacol..

[22] Shull J.D., Spady T.J., Snyder M.C., Johansson S.L., Pennington K.L. (1997). Ovary-intact, but not ovariectomized female ACI rats treated with 17 beta estradiol rapidly develop mammary carcinoma. Carcinogenesis.

[23] Blank E.W., Wong P.-Y., Lakshmanaswamy R., Guzman R., Nandi S. (2008). Both ovarian hormones estrogen and progesterone are necessary for hormonal mammary carcinogenesis in ovariectomized ACI rats. Proc. Natl. Acad. Sci. USA.

[24] Jeyabalan J., Aqil F., Soper L., Schultz D.J., Gupta R.C. (2015). Potent Chemopreventive/Antioxidant Activity Detected in Common Spices of the Apiaceae Family. Nutr. Cancer.

[25] O’day E., Lal A. (2010). MicroRNAs and their target gene networks in breast cancer. Breast Cancer Res..

[26] Munagala R., Aqil F., Vadhanam M.V., Gupta R.C. (2013). MicroRNA ‘signature’ during estrogen-mediated mammary carcinogenesis and its reversal by ellagic acid intervention. Cancer Lett..

[27] Aqil F., Jeyabalan J., Munagala R., Singh I.P., Gupta R.C. (2016). Prevention of hormonal breast cancer by dietary jamun. Mol. Nutr. Food Res..

[28] Ravoori S., Vadhanam M.V., Aqil F., Gupta R.C. (2012). Inhibition of estrogen-mediated mammary tumorigenesis by blueberry and black raspberry. J. Agric. Food Chem..

[29] Aqil F., Jeyabalan J., Munagala R., Ravoori S., Vadhanam M.V., Schultz D.J., Gupta R.C. (2017). Chemoprevention of Rat Mammary Carcinogenesis by Apiaceae Spices. Int. J. Mol. Sci..

[30] Clevenger C.V., Furth P.A., Hankinson S.E., Schuler L.A. (2003). The role of prolactin in mammary carcinoma. Endocr. Rev..

[31] Rose-Hellekant T.A., Arendt L.M., Schroeder M.D., Gilchrist K., Sandgren E.P., Schuler L.A. (2003). Prolactin induces ERalpha-positive and ERalpha-negative mammary cancer in transgenic mice. Oncogene.

[32] Ravoori S., Vadhanam M.V., Sahoo S., Srinivasan C., Gupta R.C. (2007). Mammary tumor induction in ACI rats exposed to low levels of 17 beta-estradiol. Int. J. Oncol..

[33] Li S.A., Weroha S.J., Tawfik O., Li J.J. (2002). Prevention of solely estrogen-induced mammary tumors in female aci rats by tamoxifen: Evidence for estrogen receptor mediation. J. Endocrinol..

[34] Weroha S.J., Li S.A., Tawfik O., Li J.J. (2006). Overexpression of cyclins D1 and D3 during estrogen-induced breast oncogenesis in female ACI rats. Carcinogenesis.

[35] Liehr J.G., Ricci M.J. (1996). 4-Hydroxylation of estrogens as marker of human mammary tumors. Proc. Natl. Acad. Sci. USA.

[36] Loh H.Y., Norman B.P., Lai K.S., Rahman N., Alitheen N.B.M., Osman M.A. (2019). The Regulatory Role of MicroRNAs in Breast Cancer. Int. J. Mol. Sci.

[37] Jeyabalan J., Aqil F., Munagala R., Annamalai L., Vadhanam M.V., Gupta R.C. (2014). Chemopreventive and therapeutic activity of dietary blueberry against estrogen-mediated breast cancer. J. Agric. Food Chem..

[38] Vadhanam M.V., Ravoori S., Aqil F., Gupta R.C. (2011). Chemoprevention of mammary carcinogenesis by sustained systemic delivery of ellagic acid. Eur. J. Cancer Prev..

[39] Aiyer H.S., Srinivasan C., Gupta R.C. (2008). Dietary berries and ellagic acid diminish estrogen-mediated mammary tumorigenesis in ACI rats. Nutr. Cancer.

[40] Corrales Sanchez V., Nieto-Jimenez C., Castro-Osma J.A., de Andres F., Pacheco-Linan P.J., Bravo I., Rodriguez Farinas N., Niza E., Dominguez-Jurado E., Lara-Sanchez A. (2019). Screening and Preliminary Biochemical and Biological Studies of [RuCl(p-cymene)(N,N-bis(diphenylphosphino)-isopropylamine)][BF4] in Breast Cancer Models. ACS Omega.

[41] Li L.L., He L., Wu Y.L., Zhang Y.W. (2021). Carvacrol affects breast cancer cells through TRPM7 mediated cell cycle regulation. Life Sci..

[42] Gautam N., Mantha A.K., Mittal S. (2014). Essential Oils and Their Constituents as Anticancer Agents: A Mechanistic View. Biomed. Res. Int..

[43] Ayyanar M., Subash-Babu P., Ignacimuthu S. (2013). *Syzygium cumini* (L.) Skeels., a novel therapeutic agent for diabetes: Folk medicinal and pharmacological evidences. Complement. Ther. Med..

[44] Livak K.J., Schmittgen T.D. (2001). Analysis of relative gene expression data using real-time quantitative PCR and the 2(-Delta Delta C(T)) Method. Methods.

