# Supplementary material for: Cumin Prevents 17β-Estradiol-Associated Breast Cancer in ACI Rats
Source: Int J Mol Sci. 2021 Jun 8;22(12):6194. doi: 10.3390/ijms22126194 (PMC8228322; doi:10.3390/ijms22126194)
Supplement: Supplementary file 1 [file ijms-22-06194-s001.zip › ijms-1225082-supplementary.pdf]

Supplementary Materials

Cumin Prevents 17β-Estradiol-Associated Breast Cancer in ACI Rats

Farrukh Aqil [1,2], Jeyaprakash Jeyabalan [1], Radha Munagala [1,2], Iqbal Ahmad [3], David J. Schultz [4] and Ramesh C. Gupta [1,5,*]

[1] James Graham Brown Cancer Center, University of Louisville, Louisville, KY 40202, USA; farrukh.aqil@louisville.edu (F.A.); jp3pbiotech@gmail.com (J.J.); radha.munagala@gmail.com (R.M.)
[2] Department of Medicine, University of Louisville, Louisville, KY 40202, USA
[3] Department of Ag Microbiology, Aligarh Muslim University, Aligarh 202002, India; iqbalahmad8@yahoo.co.in
[4] Department of Biology, University of Louisville, Louisville, KY 40208, USA; david.schultz@louisville.edu
[5] Department of Pharmacology and Toxicology, 580 S. Preston St, Rm 304E, Baxter II Research building, University of Louisville, Louisville, KY 40202, USA
* Correspondence: rcgupta@louisville.edu; Tel.: +502-852-3684; Fax: +502-852-3842

Supplementary Table S1. Detailed formula of the AIN93M purified diet (Catalog # TD.94048; Harlan Laboratories, Madison, WI)

Teklad Custom Research Diet Data Sheet

TD.94048 AIN-93M Purified Diet

Formula	g/Kg
Casein	**140.0**
L-Cystine	**1.8**
Corn Starch	**465.692**
Maltodextrin	**155.0**
Sucrose	**100.0**
Soybean Oil	**40.0**
Cellulose	**50.0**
Mineral Mix, AIN-93M-MX (94049)	**35.0**
Vitamin Mix, AIN-93-VX (94047)	**10.0**
Choline Bitartrate	**2.5**
TBHQ, antioxidant	**0.008**

Footnote

Reference: J. Nutr. 123:1939-1951, 1993. Formulated for the maintenance of adult, non-reproducing rodents.

Selected Nutrient Information[1]

	% by weight	% kcal from
**Protein**	12.4	13.8
**Carbohydrate**	68.4	76.0
**Fat**	4.1	10.2
**Kcal/g**	**3.6**	

[1] Values are calculated from ingredient analysis or manufacturer data

Teklad Diets are designed & manufactured for research purposes only.

Supplementary Table S2 . GC-MS Analysis - Graphics Report

Graphics Report

File : C:\HPCHEM\1\E25AMU1.D
Operator : SVKL
Acquired : 20 May 110 9:28 am using AcqMethod MANALI
Sample Name: Cumin MeOH Ext. 15/04/10 FH 110
Misc Info : 100-10-250- 3M-30-280-5M-MeIOH-HP5
Vial Number: 1

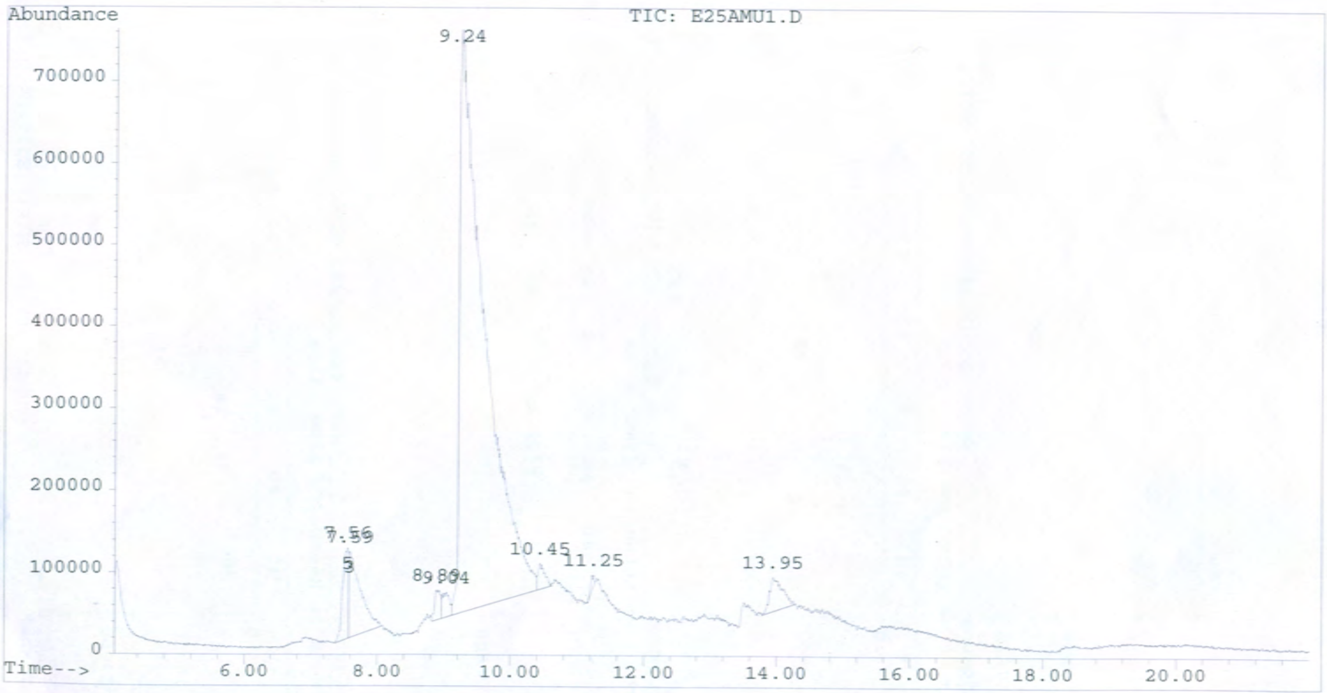

C:\HPCHEM\1\E25AMU1.D Thu May 20 11:10:02 2010 Page 1 of 3

Peak 1 at Retention Time 7.55

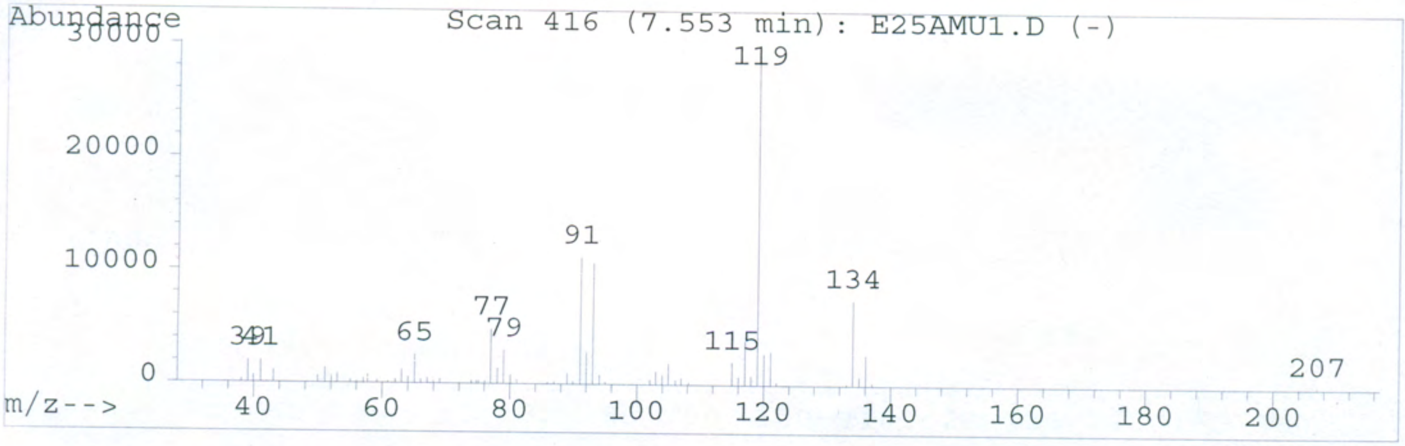

Peak 2 at Retention Time 7.59

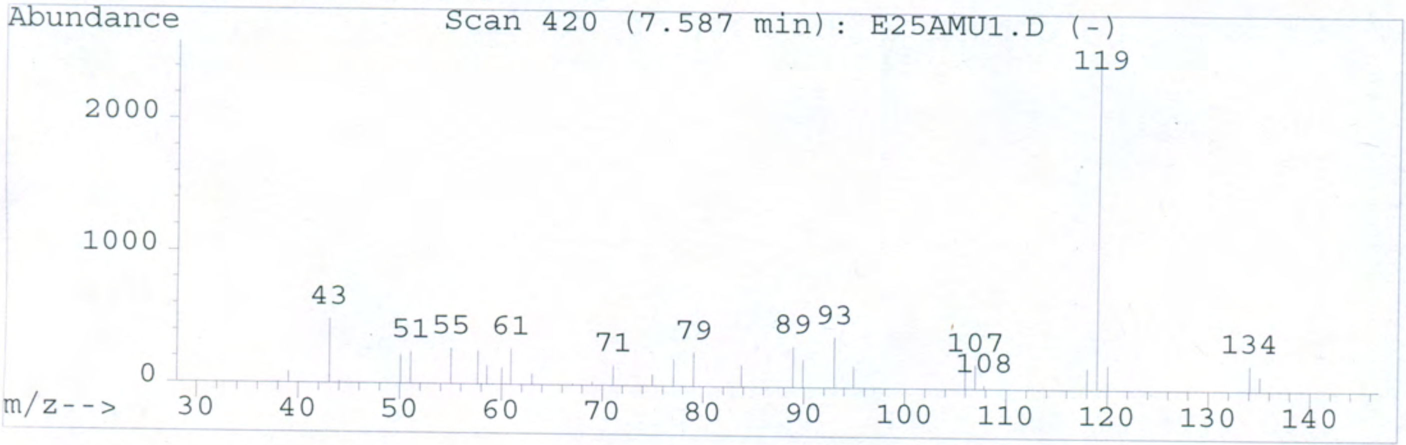

Peak 3 at Retention Time 8.89

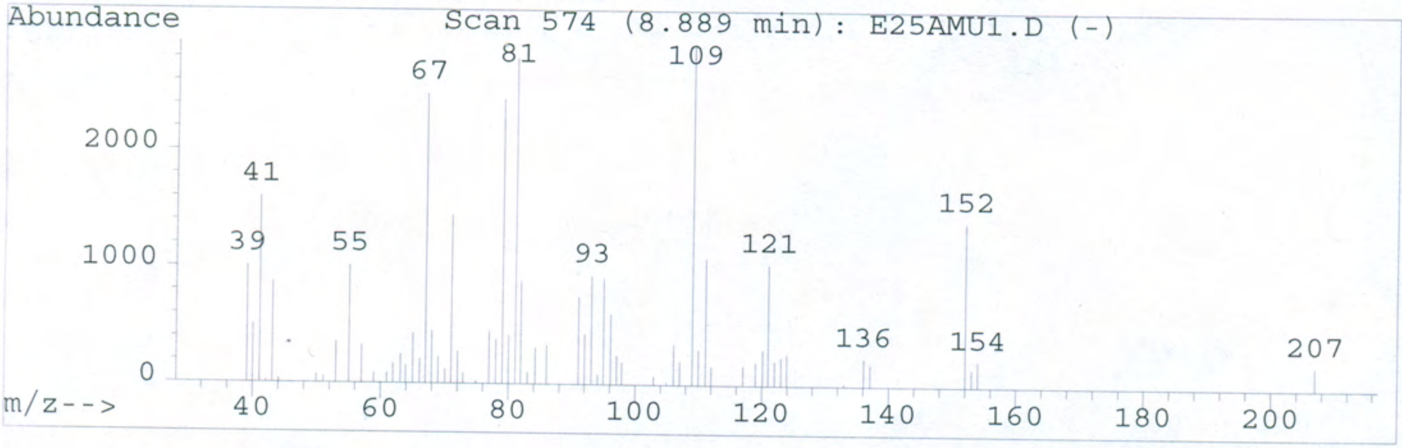

Peak 4 at Retention Time 9.04

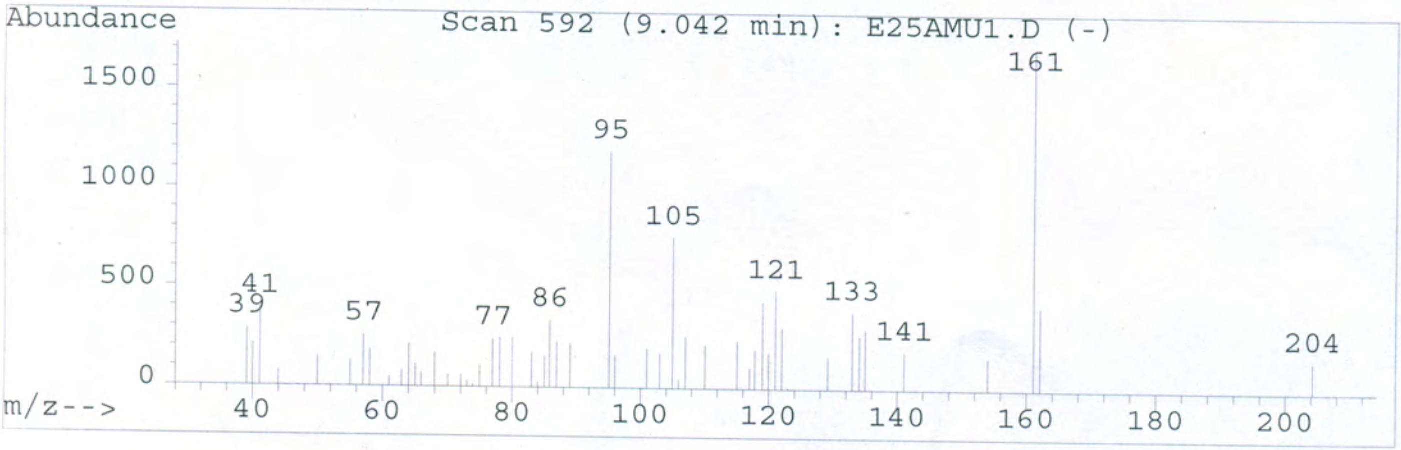

Peak 5 at Retention Time 9.24

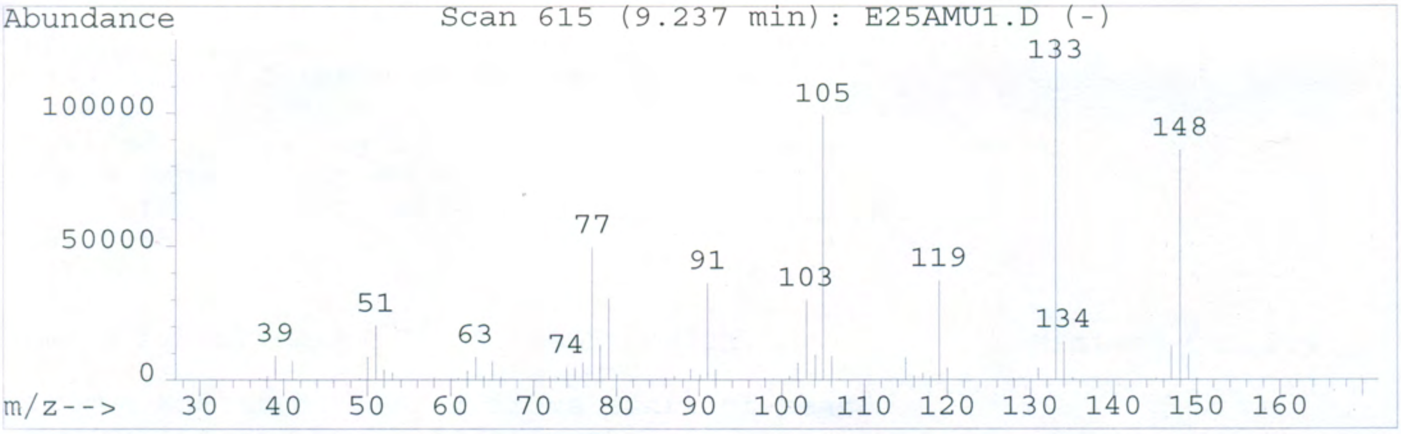

Peak 6 at Retention Time 10.44

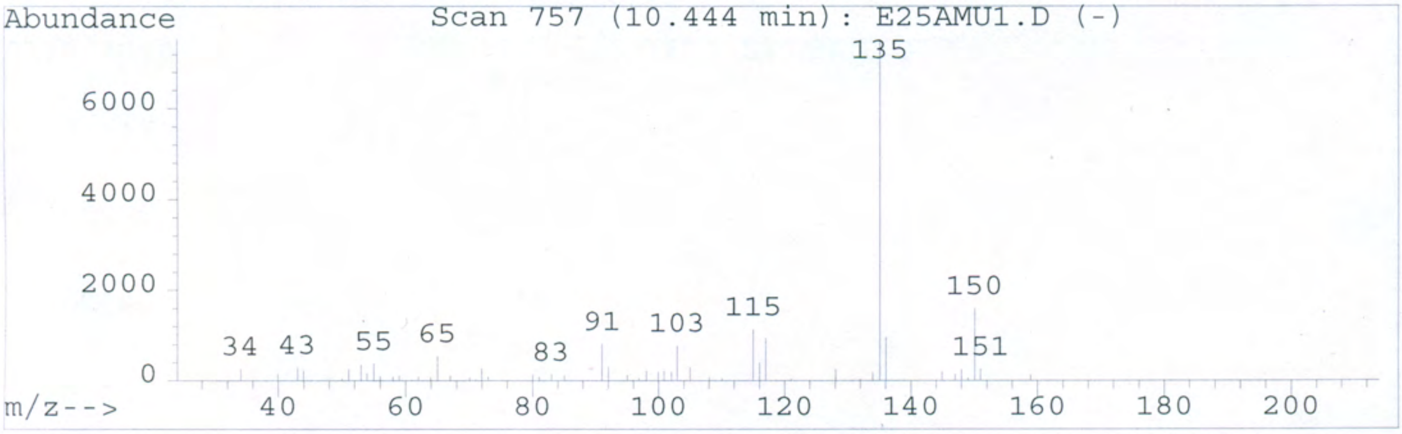

Peak 7 at Retention Time 11.25

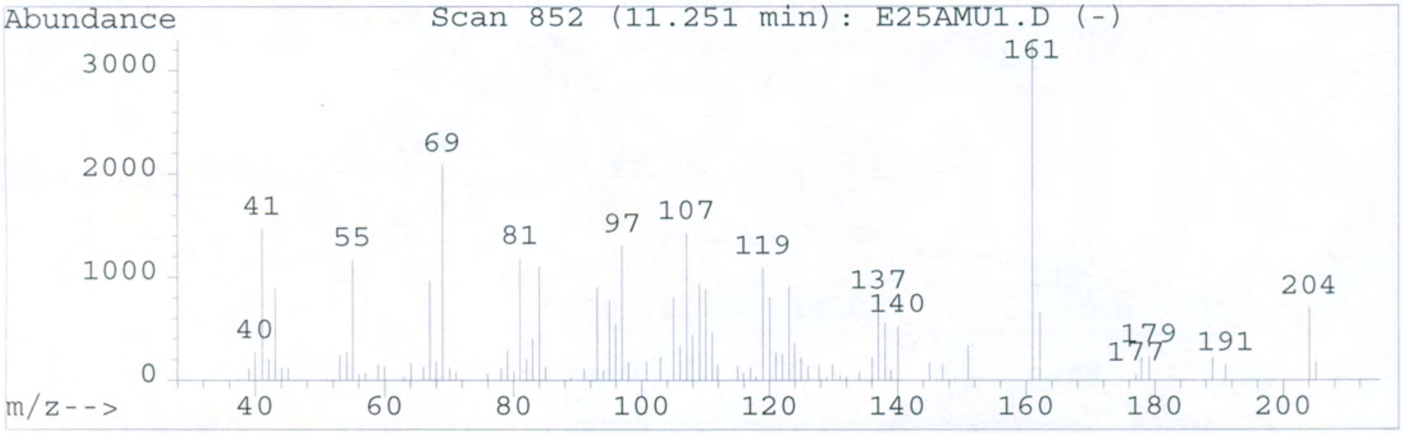

Peak 8 at Retention Time 13.95

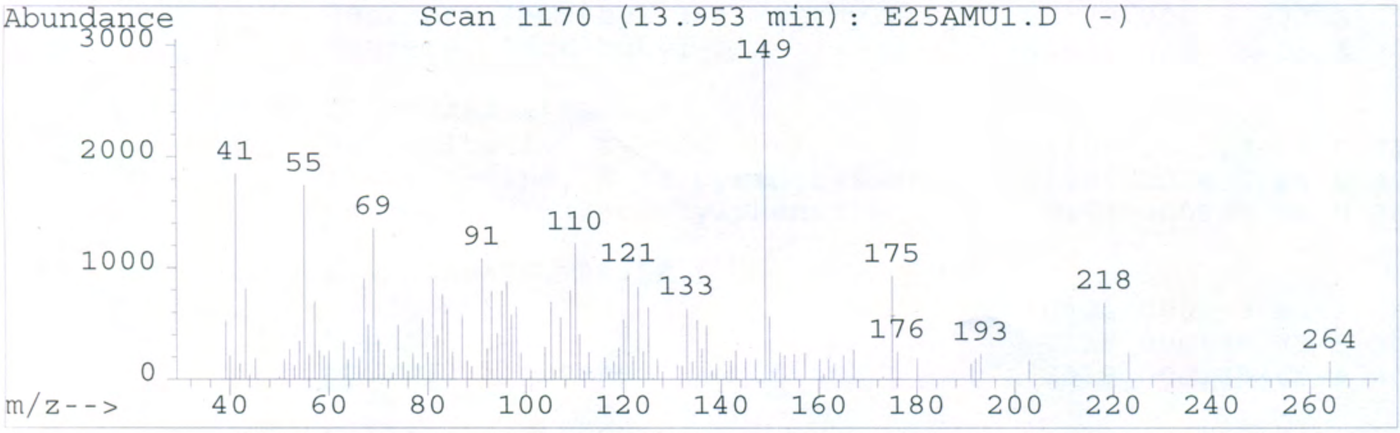

Supplementary Table S3 . Library Research Report

Summary Library Search Report

Information from Data File:
File : C:\HPCHEM\1\E25AMU1.D
Operator : SVKL
Acquired : 20 May 110 9:28 am using AcqMethod MANALI
Sample Name: Cumin MeOH Ext. 15/04/10 FH 110
Misc Info : 100-10-250- 3M-30-280-5M-MeIOH-HP5
Vial Number: 1

Search Libraries: C:\DATABASE\nbs75k.l Minimum Quality: 0

Unknown Spectrum: Apex minus start of peak
Integration Params: AutoIntegrate

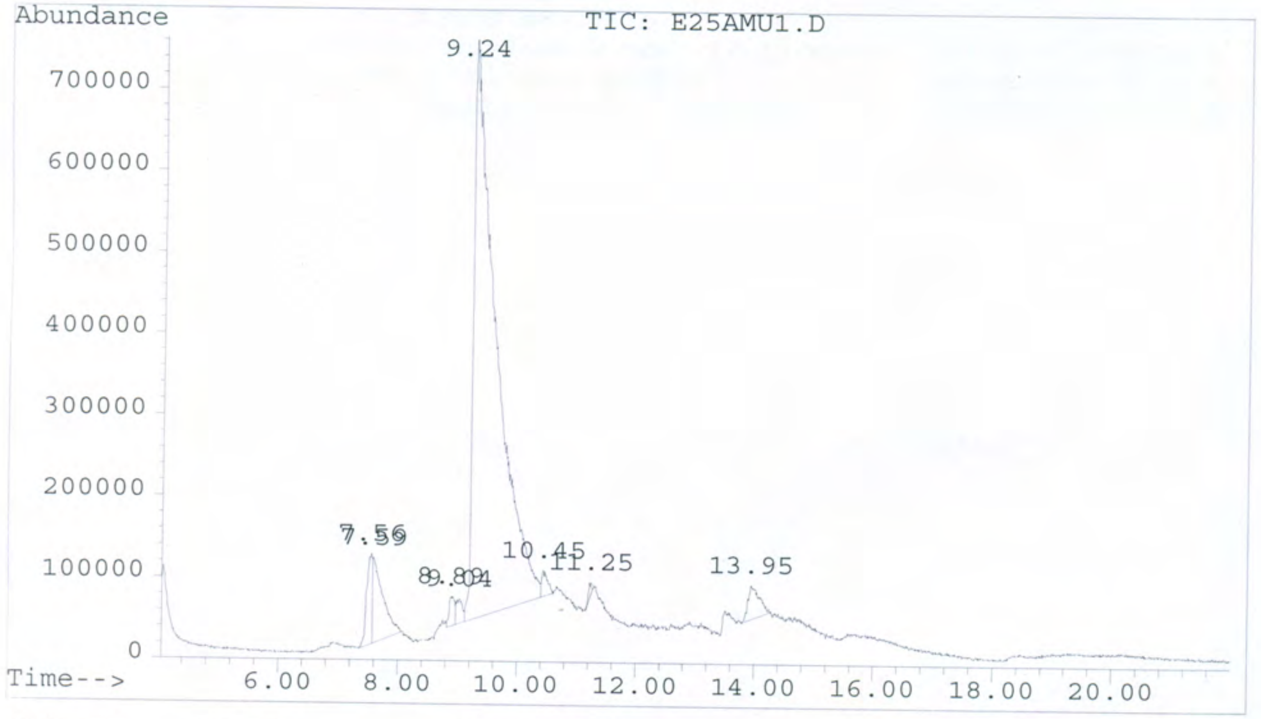

Pk#	RT	Area%	Library/ID	Ref#	CAS#	Qual
1	7.55	3.07	C:\DATABASE\NBS75K.L			
			Benzene, 1-methyl-4-(1-methylethyl	65539	000099-87-6	92
			Benzene, 1-methyl-3-(1-methylethyl	65579	000535-77-3	70
			Benzene, tert-butyl-	65529	000098-06-6	70
2	7.59	5.99	C:\DATABASE\NBS75K.L			
			Benzonitrile, 4-hydroxy-	64499	000767-00-0	47
			1-Butanamine, N-(2-pyridinylmethyl	12959	007032-24-8	40
			Ethanone, 1-(3-methylphenyl)-	6153	000585-74-0	38
3	8.89	1.04	C:\DATABASE\NBS75K.L			
			Pulegone	10406	000089-82-7	46
			Mequinol	4138	000150-76-5	30
			Spiro[2.5]octane	2339	000185-65-9	30
4	9.04	1.03	C:\DATABASE\NBS75K.L			
			1H-Cyclopropa[a]naphthalene, 1a,2,	23897	017334-55-3	47
			3a,7-Methano-3aH-cyclopentacyclooc	23952	000469-92-1	43
			2-Hexen-1-one, 1-(2-hydroxy-5-meth	23838	051956-79-7	35

Pk#	RT	Area%	Library/ID	Ref#	CAS#	Qual
5	9.24	85.11	C:\DATABASE\NBS75K.L			
			Benzaldehyde, 4-(1-methylethyl)-	66596	000122-03-2	97
			Ethanone, 1-(2,4-dimethylphenyl)-	66595	000089-74-7	91
			Ethanone, 1-(2,5-dimethylphenyl)-	9319	002142-73-6	87
6	10.44	1.03	C:\DATABASE\NBS75K.L			
			Thymol	66816	000089-83-8	74
			Phenol, 2-methyl-5-(1-methylethyl)	66779	000499-75-2	72
			Phenol, p-tert-butyl-	9864	000098-54-4	58
7	11.25	0.24	C:\DATABASE\NBS75K.L			
			Carotol	28216	000465-28-1	53
			Naphthalene, 1,2,3,4,4a,5,6,8a-oct	23957	039029-41-9	50
			Germacrene D	23911	023986-74-5	49
8	13.95	2.51	C:\DATABASE\NBS75K.L			
			Trichothec-9-en-8-one, 12,13-epoxy	36571	002199-06-6	32
			Silane, chlorotripropyl-	20645	000995-25-5	27
			2(1H)-Naphthalenone, octahydro-1,4	17896	022738-31-4	25

Supplementary Table S4 . Area Percent Report

Area Percent Report -- Sorted by Signal

Information from Data File:
File : C:\HPCHEM\1\E25AMU1.D
Operator : SVKL
Acquired : 20 May 110 9:28 am using AcqMethod MANALI
Sample Name: Cumin MeOH Ext. 15/04/10 FH 110
Misc Info : 100-10-250- 3M-30-280-5M-MeIOH-HP5
Vial Number: 1
CurrentMeth: C:\HPCHEM\1\METHODS\MANALI.M

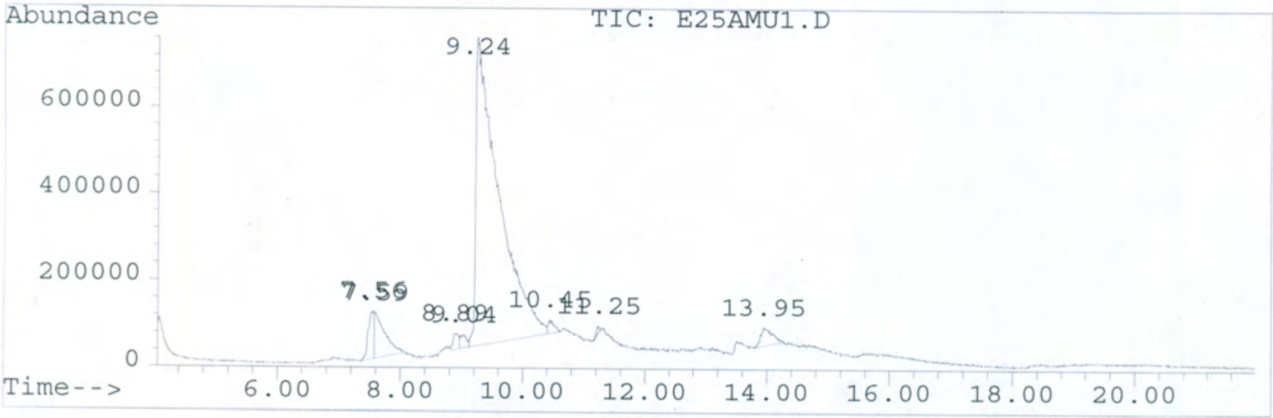

Retention Time	Area	Area %	Ratio %	Type	Width
Total Ion Chromatogram					
7.556	6641441	3.065	3.602	PV	0.101
7.587	12979705	5.991	7.039	VV	0.207
8.891	2244180	1.036	1.217	VV	0.082
9.041	2223002	1.026	1.206	VV	0.125
9.242	184390447	85.106	100.000	VV	0.321
10.446	2222522	1.026	1.205	VV	0.106
11.253	520542	0.240	0.282	BV	0.055
13.951	5437390	2.510	2.949	PB	0.170

Supplementary Table S5. Allometric dose calculations based on body surface area

Average feed intake/rat/day = 10 g; Cumin extract dose/rat/day @1% (w/w) supplement = 100 mg; Average body weight of female ACI rat = 200 g (0.2 kg); Intake of dried extract diet (mg/kg body weight) @ 1% = 100 mg/0.2 kg = 500 mg

$$\text{Human Eq. Dose (mg/kg)} = \text{Animal dose (mg/kg)} \times \frac{\text{Animal Km (6 for rats)}}{\text{Human Km (37 for 60 kg adult)}}$$

$$= 500 \times \frac{6}{37}$$

$$= 81 \text{ mg (equivalent of 0.63 g of cumin seeds)}$$

where *Km factor* is the body weight (kg) divided by body surface area (m^2)

Reference: Reagan-Shaw S, Nihal M, Ahmad N (2008) Dose translation from animal to human studies revisited. FASEB J 22:659–661
